# The Use of Primary Spiral Ganglion Cells in Studying Glutamate Receptor Function and Excitotoxicity in the Cochlea

**DOI:** 10.3390/cells15090777

**Published:** 2026-04-25

**Authors:** Eugenue V. Polikarpov, Elena A. Smolyarchuk, Andrey P. Fisenko, Zanda V. Bakaeva

**Affiliations:** 1 Department of Pharmacology, Institute of Pharmacy, Sechenov First Moscow State Medical University (Sechenov University), 119991 Moscow, Russia; 2 National Medical Research Center of Children’s Health, 119991 Moscow, Russia

**Keywords:** spiral ganglion neurons, spiral ganglion cells, glutamate excitotoxicity, primary cell culture, sensorineural hearing loss, neurotransmission, coclear synaptopathy

## Abstract

Sensorineural hearing loss (SNHL) can result from genetic mutations, excessive noise exposure, ototoxic drugs, and aging. Glutamate excitotoxicity is one of the underlying mechanisms of SNHL. However, the specific roles of different glutamate receptor subtypes in normal signaling and excitotoxic damage remain unclear. Addressing these questions requires relevant experimental models. This review compares existing protocols for the isolation and cultivation of primary spiral ganglion cells. It also evaluates the utility of this model for studying glutamatergic transmission and glutamate-induced excitotoxicity. A literature search was conducted in PubMed, Scopus, Google Scholar, and Web of Science. We identified 16 relevant English-language articles published since 1990, when the model was first used to study glutamatergic signaling. Our analysis reveals significant heterogeneity in spiral ganglion cell isolation protocols and culture conditions. We highlight major differences in glutamate concentrations and exposure times used to model excitotoxicity. The most significant limitation of this model is the loss of the native microenvironment of auditory neurons, including their dendritic and axonal contacts. Nevertheless, primary spiral ganglion cells serve as a suitable in vitro model for investigating auditory neuron function and pathology. The number of neurons and neurite length serve as reliable indicators of otoprotective effects under conditions of glutamate excitotoxicity. Based on an analysis of the key stages of primary SGC culture establishment, this study proposes approaches to overcome limitations and improve the practice of using this model. A better understanding of the function of glutamate receptors of SGNs and the mechanisms behind glutamate excitotoxicity could help us to develop new treatments for SNHL. This review serves as a practical guide for researchers implementing or optimizing primary SGC cultures.

## 1. Introduction

Sensorineural hearing loss (SNHL) is the most common type of hearing impairment. It is characterized by a loss of hair cells and/or damage to the auditory nerve. In the world, more than 5% of the population suffers from hearing loss [1]. Factors that cause hearing loss can be categorized as genetic and non-genetic (acquired) [2]. One of the major non-genetic factors for hearing loss is acoustic trauma, which can result from both sudden and prolonged exposure to loud sounds [3]. Hearing loss is often associated with the use of ototoxic drugs, such as aminoglycoside antibiotics and platinum-based chemotherapeutic agents like cisplatin and carboplatin. Other drugs known to have ototoxic effects include loop diuretics, macrolides (e.g., azithromycin), antimalarial drugs (e.g., chloroquine and hydroxychloroquine) and NSAIDs (e.g., acetylsalicylic acid) [4]. Another significant factor in hearing loss is age-related degeneration of the sound-perceiving and sound-conducting apparatus of the auditory system [5].

In mammals, degeneration of sensory hair cells (HCs) and spiral ganglion neurons (SGNs) is irreversible, and a deficit in either of them can lead to permanent hearing loss [6]. Current hearing rehabilitation approaches, such as hearing aids and cochlear implants, can only partially restore hearing function. However, the quality of sound perceived through these devices does not replicate normal hearing [7,8].

Type I SGNs account for 90–95% of the SGC population. They are responsible for transmitting afferent signals from the inner hair cells to the brain [9]. Excitatory transmission at the inner hair cell/type I SGN synapse (IHC/SGN synapse) is mediated by glutamate. The presynaptic terminals of inner hair cells release glutamate onto the postsynaptic terminals of type I SGN dendrites [10,11]. The postsynaptic terminals of these neurons express all classes of glutamate receptors, notably α-amino-3-hydroxy-5-methyl-4-isoxazolepropionic acid receptors (AMPARs), N-methyl-D-aspartate receptors (NMDARs), kainate receptors (KARs) and metabotropic receptors (mGluRs) [12,13,14]. Since AMPARs have fast kinetics, they are believed to play a key role in neurotransmission at the IHC/SGN synapse. In contrast, NMDARs, which are characterized by their slow kinetics, are thought not to be directly involved in neurotransmission at this synapse [15,16,17,18]. By comparison, NMDARs play a crucial role in neurotransmission within the central nervous system. At negative membrane potentials, NMDARs experience a magnesium block. This block can be removed through an initial depolarization of the membrane, which is mediated by AMPARs [19].

AMPARs are tetrameric ligand-gated ion channels that can be composed of various combinations of GluA1, GluA2, GluA3 and GluA4 subunits in either homomeric or heteromeric arrangements. They exhibit varying degrees of permeability to sodium, potassium and calcium ions. The presence of the GluA2 subunit typically renders the channel less permeable to calcium ions. It has been noted that during cochlear development, the expression of calcium-permeable AMPARs (CP-AMPARs) lacking the GluA2 subunit is suppressed. They suggest that in the mature cochlea, glutamatergic transmission is facilitated by AMPARs consisting of GluA2, GluA3 and GluA4 subunits. Current evidence suggests that afferent fibers in the mature cochlea contain both GluA2-lacking CP-AMPARs and GluA2-containing calcium-impermeable AMPAR (CI-AMPARs) [17,20,21,22].

Exposure to acute or chronic noise, cochlear ischemia, or ototoxic drugs leads to excessive glutamate release from the inner hair cells into the synaptic cleft. High levels of glutamate can cause overexcitation and the destruction of type I SGN dendrites [23]. It has been demonstrated that one of the key components mediating the effects of glutamate excitotoxicity in adult animals is the CP-AMPAR [21]. Excessive excitation of type I SGN terminals leads to the accumulation of Ca^2+^ and subsequent swelling of the terminals [20]. The glutamatergic agonist induces morphological changes similar to those seen after sound overexposure. Applying antagonists of CP-AMPARs prevents excitotoxic damage to afferent terminals [20,21]. Despite data indicating the presence of CP-AMPARs in the mature cochlea, their role in the development of glutamate excitotoxicity and prevalence in the mature inner ear are not yet fully understood. By comparison, glutamate excitotoxicity in the central nervous system is primarily caused by the overactivation of NMDARs [24]. This leads to a significant increase in the concentration of intracellular calcium. Intracellular processes are initiated that result in the excessive generation of reactive oxygen species (ROS), mitochondrial damage, and the activation of caspases and other pro-apoptotic factors [25,26,27].

The role of NMDARs in auditory neurons is still the subject of discussion. The molecular heterogeneity of NMDAR channels provides their dual role in physiological and pathological functions [28]. Previous studies show that combined application of NMDA and glycine receptor agonists in a magnesium-free solution does not produce a significant postsynaptic potential or alter calcium concentration in auditory neurons [15,16,29,30]. However, Wang et al. revealed that in isolated auditory neurons from newborn mice, intracellular calcium levels increase in response to NMDA when D-serine is present as a co-agonist, whereas no such increase occurs with glycine [18]. Therefore, it is reasonable to propose that D-serine plays a key role in modulating the function of NMDARs in the cochlea in both normal physiological processes and pathological states. It is assumed that NMDARs contribute to noise- and salicylate-induced tinnitus. Research suggests that NMDARs may be involved in gentamycin-induced ototoxicity [31]. They also play a role in noise-induced damage to auditory neuron afferents and presbycusis [32,33,34,35]. The specific types of receptors, the downstream mechanisms that cause damage, and the factors that contribute to the selective vulnerability of certain synapses are not yet fully understood.

Given the dual role of the NMDARs, it is possible that they regulate the expression of the AMPARs to modulate synaptic transmission [36,37]. They may also contribute to neuritogenesis and synaptogenesis, as well as promote the survival of SGNs in the developing cochlea [38,39].

Several studies have demonstrated the presence of mGluRs in the cochlea and their functional significance in auditory processing. The eight identified mGluRs subtypes are classified into three groups: Group I (mGluR1 and mGluR5); Group II (mGluR2 and mGluR3); and Group III (mGluR4, mGluR6, mGluR7 and mGluR8). The cochlea has been found to express members of all three groups of metabotropic glutamate receptors [40,41]. However, the cochlear function of individual mGluR subtypes is still poorly defined. It is believed that mGluRs have slow kinetics. Consequently, they are not directly involved in fast excitatory neurotransmission in the cochlea but rather modulate this process [42]. Experimental data suggested that mGluRI, localized on the postsynaptic membrane, modulates the excitability of SGNs. This leads to an enhanced response to intense stimulation, resulting in increased cochlear vulnerability to noise-induced trauma and glutamate excitotoxicity. Therefore, mGluR1 inhibitors could be a promising therapeutic approach for treating SNHL [13].

According to Friedman et al. [43], genetic variations in GRM7, a gene encoding mGluR7, likely influence age-related hearing impairment (ARHI) by altering susceptibility to glutamate excitotoxicity in auditory neurons. It is proposed that mGluR7 receptors, localized on the presynaptic membrane, act as autoreceptors by regulating glutamate concentration in the synaptic cleft through control of its release and preventing excessive glutamate accumulation [44,45].

Glutamate excitotoxicity is widely recognized as playing a central role in the mechanisms underlying noise-induced synaptopathy [46,47,48,49]. It has long been thought that the primary factor in the pathogenesis of noise-induced damage and age-related hearing loss is the loss of hair cells and SGNs. Decades of research have demonstrated that cochlear nerve synapses can degenerate even when hair cells and SGNs remain structurally intact. Research using animal models has demonstrated that moderate noise exposure initially results in the loss of synaptic connections between inner hair cells and their associated neurons. The subsequent delayed death of neurons is linked to the loss of connectivity with inner hair cells, which provide essential neurotrophic support for maintenance of the cochlear innervation [46]. The mechanism underlying the development of noise-induced synaptopathy is not fully understood. It is believed that excessive activation of CP-AMPARs by glutamate leads to damage to cochlear ribbon synapses [20,21]. It is suggested that one mechanism of synaptopathy involves noise-induced disruption of glutamate release and uptake-related protein expression [47]. Pure-tone audiometry is unable to detect this type of hearing impairment, as hearing thresholds may remain within the normal range. Current clinical diagnostic methods lack the capability to identify diffuse synaptic damage between the inner hair cells and the auditory nerve fibers. For this reason, cochlear synaptopathy is often referred to as “hidden hearing loss”.

Primary neurons under in vitro conditions are a well-established experimental model, as evidenced by a significant number of studies published using primary neurons of the brain, spinal cord, gastrointestinal tract, and other sources [50,51,52,53,54]. In auditory research, primary cultures of spiral ganglion cells (SGCs) have served as a valuable model for many years. This review provides examples of using SGCs isolated from the modiolar tissue of adult, neonatal, and embryonic animals to investigate glutamatergic transmission and excitotoxicity. This review provides examples of using SGCs isolated from the modiolar tissue of adult, neonatal, and embryonic animals to investigate glutamatergic transmission and excitotoxicity. Analysis of the presented studies can assist researchers in reproducing this model in their own laboratories, building upon the accumulated experience from previous work.

## 2. Literature Search Strategy

We present and analyze the main results on studying glutamatergic transmission and excitotoxicity using primary SGCs. A literature search was conducted using PubMed, Scopus, Google Scholar, and Web of Science databases. Reference lists of included studies were manually screened to identify additional eligible publications not retrieved by the electronic search. We included in our analysis only English-language publications with accessible full text. Original articles and relevant narrative reviews were considered eligible. Our review analyzes articles from 1990 onward, when the primary SGC model in vitro was first used to investigate the glutamatergic system [55]. We extracted data from dose–response curves presented in the figures using WebPlotDigitize (version 4.8)

## 3. Results

SGNs have a bipolar morphology in vivo. Type I SGNs form glutamatergic synaptic contacts with inner hair cells through their dendrites and transmit information to the brainstem via their axons. The neurotransmitter is released from inner hair cells onto the distal end of the dendrite at the IHC-SGN synapse. In culture, primary SGCs grown as a monolayer lack the natural microenvironment and specific intercellular signaling present in vivo [56]. This limitation alters the morphology, gene expression, and, consequently, the physiology of auditory neurons. It is important to note that cell isolation from modiolar tissue results in a heterogeneous population of primary cells. This includes not only SGNs, but also other cell types, such as Schwann cells, satellite glia and fibroblasts. Therefore, in this context, cells derived from the modiolus are commonly referred to as SGCs in the literature. The study of the glutamatergic system in isolated SGCs is conducted using two main approaches: primary cell cultures and experiments on acutely isolated (non-cultured) cells. Acutely isolated (non-cultured) cells serve as the primary model for functional assays, such as patch-clamp and calcium imaging, in the studies covered in this review. Studies investigating glutamate excitotoxicity using primary SGC culture have typically included assessments of cellular morphology, viability, and the expression levels of pro- and anti-apoptotic factors and oxidative stress markers. Most studies on primary SGCs have used samples from newborns. This may be due to the difficulties involved in obtaining adult neurons.

The process of obtaining primary SGCs involves several stages. This analysis reviews the procedures described in various studies for obtaining primary SGCs, including the isolation of modiolus tissue and cell dissociation techniques, as well as the different methods available for culturing SGCs. Acutely isolated (non-cultured) cells either bypass the culturing step entirely or undergo only several hours of short-term culturing. Unlike cultured SGCs, acutely isolated SGCs are typically transferred directly into experimental solutions for immediate use. Existing protocols for obtaining and cultivating primary SGCs vary across different studies. This can affect not only the survival and development of neurons but also the reproducibility of research methods utilizing these primary cultures. All procedures, including the dissection of the cochlea, preparation of reagents for cell dissociation and cultivation of primary cell cultures, must be conducted under sterile conditions. SGCs are highly sensitive to disturbances, so all steps are carried out with great care.

### 3.1. Cochlea Extraction and Modiolus Tissue Isolation

It is not described in the literature how methodological specifics at this stage impact experimental outcomes in cochlear sample data in studies of glutamatergic effects. Thus, this section reviews the fundamental principles and procedures of cochlear extraction across different experimental contexts. The main parameters of this stage are the speed of cochlear isolation and surface dissection. The interval between animal decapitation and the placement of cochlear samples into the incubation or experimental medium should be kept as short as possible. These procedures require substantial practical experience and a refined technique in order to minimize the risk of damaging the sample [57].

Numerous articles describe methods for the extraction and microdissection of the cochlea of experimental animals at various ages [58,59,60,61,62,63,64,65,66]. However, specimens from the cochlea obtained during the prenatal, neonatal and early postnatal periods are more frequently used for isolating SGCs. The bony labyrinth of newborn rodents can easily be removed without damaging the underlying tissues because it has not yet undergone ossification. At the onset of postnatal day 3 (P3), the spiral lamina and the cartilage within the cochlea begin to ossify. Caution must be exercised when removing the bony capsule, spiral ligament, stria vascularis and organ of Corti, as neuronal cell bodies may detach from the modiolus along with the excised tissues. These procedures are performed using ultrafine forceps and a microdissection knife. After P14, the removal of the completely ossified bony capsule of the bony labyrinth often damages the soft tissues of the cochlea, making it difficult to obtain intact samples for in vitro culture establishment [57,67].

The dissection of the cochlea and collection of the modiolar portion containing SGNs within the spiral lamina are performed in a balanced saline solution. For this purpose, ice-cold phosphate-buffered solution [68,69], or Hank’s Balanced Salt Solution (HBSS) without calcium and magnesium ions, is most commonly used [70,71].

### 3.2. Dissociation

Modiolar tissue dissociation is achieved through a multistep procedure combining mechanical disruption and enzymatic digestion. Mechanical dissociation involves carefully pipetting the modiolar tissue to achieve a homogeneous solution, aiming for maximum cell dissociation. However, mechanical dissociation can result in cell loss, so the duration and intensity of pipetting must be carefully controlled to minimize damage. In the process of dissociation, auditory neurons lose their neurites. After cell seeding, neuritogenesis is triggered. Neurons regain their bipolar shape, but some neurons may acquire multipolar and unipolar shapes [72].

Enzymatic disaggregation of SGCs typically involves one or a combination of enzymes. The conditions of enzymatic dissociation, including the composition of the digestive enzyme solution used and duration of exposure, can have a significant impact on cell viability and various cellular functions. Table 1 summarizes the enzymatic dissociation protocols used in key studies investigating the glutamatergic system in primary SGNs.

Since most dissociation modes involve trypsin, the dissection solution is most often HBSS without calcium and magnesium, or PBS. Balanced buffer solutions provide a physiological environment with stable osmolarity and pH. Trypsin is the most commonly used enzyme to break down cell adhesion components. Collagenase, when used in combination with trypsin, is particularly essential for effectively isolating SGCs embedded in the collagen-rich tissue of the modiolus. In studies investigating the effects of glutamate on SGNs in primary culture, the enzymatic dissociation step mostly involves a combination of trypsin and collagenase in the digestive solution. Additionally, DNase is frequently included in the dissociation process to cleave DNA strands released from dead cells. This prevents cell aggregation and facilitates the release of individual cells into a homogeneous suspension.

Enzymatic dissociation has the potential to alter gene expression and disrupt signaling pathways in cells [73,74]. Therefore, it cannot be ruled out that the process of isolating and dissociating SGCs can significantly alter the expression profile of SGNs’ glutamate receptors. Such changes could affect the cellular response to glutamate exposure in an experiment.

**Table 1:** Conditions for enzymatic dissociation included in protocols for studying glutamatergic system on primary SGCs. Not reported data are presented by the symbol “NA”.

Study	Animal Species and Age	Enzyme Type	Enzyme Concentration	Units	Duration [min]	Temperature [°C]
Yamaguchi and Ohmori, 1990 [55]	Chick embryo (16–19 embryonic days)	Collagenase Papain	0.3 10	units/mL units/mL	30 25	37
Lefebvre et al., 1991 [75]	Rats (P5)	Trypsin Collagenase DNAse	0.1 0.1 0.01	% % %	25	37
Lefebvre et al., 1991 [75]	Adult rats	Collagenase Trypsin	5 0.1	% %	20 17	37
Nakagawa et al., 1991 [29]	Adult guinea pigs	Collagenase or Dispase	0.5 500	mg/mL IU/mL	30–60 30	31
Nakagawa et al., 1991 [29]	Chickens from 2 to 5 wk post-hatch of either sex	Collagenase Trypsin type IX or Dispase	1.0 0.5 1000	mg/mL mg/mL IU/mL	30 30 60–90	31
Harada et al., 1994 [76]	Adult guinea pigs	Collagenase	0.1	mg/mL	30	NA
Shimozono et al., 1995 [30]	Adult guinea pigs	Collagenase or Dispase	0.5 500	mg/mL IU/mL	30–60 30	31
Peng et al., 2004 [13]	Mice (P0, adult)	Collagenase type IV Trypsin	0.5 2.5	mg/mL mg/mL	30 + 30	On ice 37
Zhai et al., 2004 [70]	Mice (P3)	Collagenase Trypsin	0.25 0.25	% %	25	37
Chen et al., 2007 [36]	Mice (P6–P8)	Collagenase type IV Trypsin	0.5 2.5	mg/mL mg/mL	25	37
Chen et al., 2009 [37]	Mice (P3–P5)	Collagenase type IV Trypsin	0.5 2.5	mg/mL mg/mL	25	37
Xiao et al., 2010 [77]	Rats (P3–P7)	Trypsin	NA	NA	8 + 6	37 low temperature
Ding et al., 2015 [78]	Rats (P0–P3)	Trypsinase	0.125	%	15	37
Bai et al., 2016 [79]	Rats (P < 5)	Trypsinase	0.125	%	15	37
Li et al., 2018 [80]	Rats (embryonic day-18)	Trypsin	1.25	mg/mL	10	37
Sun et al., 2021 [81]	Rats (P3)	Collagenase type IV Trypsin	0.1 0.25	% %	20	37
Wang et al., 2021 [18]	Rats (P1–P4)	Collagenase type IV Trypsin	0.5 2.5	mg/mL mg/mL	60	37

### 3.3. Culture Maintenance

Some factors can limit the accuracy and representativeness of results obtained from primary SGCs. To study the glutamatergic system in primary SGC culture, conditions must allow the neurons to respond normally to glutamate receptor agonists and antagonists. We propose that this sensitivity depends on the expression levels and subunit composition of glutamate receptors, which are critically influenced by the culture conditions in vitro. Selecting the appropriate culture medium and coating substrate is essential for mimicking physiological conditions in vitro and ensuring successful cell cultivation.

The methodological heterogeneity in primary SGC cultivation, as presented in Table 2, reflects the absence of established guidelines for substrate selection in glutamatergic system studies. However, SGNs demonstrate their viability when using various coating substrates, such as collagen [70], poly-L-lysine [82], poly-D-lysine [83,84], laminin [85], and poly-ornithine [18,86,87]. Commercially available cell adhesives are also utilized [30,76,79,88]. There is no consensus regarding the choice of coating substrate.

Primary cultures of SGCs are established using various media formulations. Research protocols examining glutamatergic effects on SGCs implement both serum-free and serum-containing culture. Standard modified Dulbecco’s Modified Eagle Medium (DMEM) and DMEM/F12 serve as the basal medium in most protocols, which is then supplemented with a range of additives such as serum, neurotrophins, growth factors, and specialized serum-free supplements B-27 and N-2 [77,81,89]. Most protocols use serum-containing media.

It is important to note that the antioxidant components of the B-27 supplement—such as vitamin E, vitamin E acetate, superoxide dismutase, catalase, and glutathione—enhance neuronal survival by reducing oxidative stress in cells [86]. Culture media typically contain amino acids, inorganic and organic ions, hormones, growth factors, as well as antibiotics and antifungals. These components help maintain optimal osmolarity, pH, viscosity, and the required levels of CO_2_ and oxygen. Primary cultures of SGCs are heterogeneous and consist not only of neurons but also other cell types, such as Schwann cells, satellite glia and fibroblasts [49,68]. In primary cell cultures derived from the cochlea of developing animals, there is an active proliferation of non-neuronal components, which poses challenges, particularly during prolonged cultivation. For this reason, most studies have only permitted short-term culture of SGC populations (2–5 days) [68]. Thus, the pre-experimental cultivation time is a critical parameter. The significant presence of non-neuronal cells inevitably influences the experimental outcomes. Several studies culturing SGCs mention the use of Neurobasal Medium (NBM) [83,90]. NBM is widely used to support neuronal growth and development in vitro. Initially, this culture medium was developed for the optimal cultivation of hippocampal neurons [91]. One of the advantages of NBM over other media is its ability to promote high neuronal survival rates while reducing the proliferation of glial cells in cultures [92]. Only one study investigating the effects of the glutamatergic system mentions the use of NBM supplemented with B27 for a 5-day culture period [77]. That culture exhibited a high proportion of neurons and good viability. The cell culture was predominantly composed of neurons with a normal bipolar morphology. We suggest that using neurobasal medium could be a promising approach for optimizing the protocol to obtain primary SGC cultures.

Cellular heterogeneity in primary cultures presents a challenge for techniques like immunoblotting. Observed changes in protein expression reflect the entire mixed population rather than neurons specifically [93]. Effective suppression of glial cell and fibroblast growth is achieved by adding the mitotic inhibitors like cytosine β-D-arabinofuranoside (Ara-C). Its mechanism of action is based on the disruption of DNA synthesis. Schwieger et al. demonstrated that the addition of 5 μM Ara-C allows for the prolongation of cultivation time up to one week and increases the ratio of neurons to non-neuronal cells [68]. When using cochleae from newborn animals, this approach may be necessary due to the presence of rapidly dividing stem cells. In the study by Xiao et al. [77], which examined quinolinic acid-mediated effects, the authors reported using Ara-C. They noted that culture purity reached 95% SGNs.

The morphology of SGNs in vivo is typically bipolar, with dendrites connecting to inner hair cells and axons projecting to the cochlear nuclei in the medulla oblongata. SGNs require the maintenance of synaptic contacts with both their peripheral and central targets to ensure survival. Presynaptic cells can offer trophic support through the release of neurotrophic factors, neurotransmitters and by causing depolarization due to synaptic activity [79]. It is believed that the additive action of multiple neurotrophins plays a significant role in maintaining neuron survival, synaptogenesis and their normal bipolar morphology [94]. Cochlear nucleus neurons can supply postsynaptic trophic support [95]. In vitro, SGNs lose afferent stimulation and trophic support from supporting cells, hair cells, and cochlear nucleus cells. To compensate for this deficit, adding neurotrophic factors to the culture medium is essential. Studies have shown that the neurotrophin family members brain-derived neurotrophic factor (BDNF), neurotrophin-3 (NT3), neurotrophin-4/5 (NT-4/5) and the neuropoietic cytokines ciliary neurotrophic factor (CTNF), leukemia inhibitory factor (LIF) can significantly impact neuronal survival and neurotogenesis in vitro [75,83,84,96,97]. Neurotrophic factors are potent modulators of the activity and expression of ion channels and receptors [98]. Therefore, using a combination of these factors is rational. It has been demonstrated that neurotrophic factors, when combined with depolarization induced by veratridine or elevated potassium concentrations in the medium, enhance the survival of SGNs in primary culture in an additive manner [87].

As the primary excitatory neurotransmitter, glutamate likely exerts a wide range of effects beyond neurotransmission, similar to its role in the central nervous system [99]. Neuronal maturation during development requires both trophic support and afferent-induced synaptic activity. Glutamatergic stimulation is intimately involved in these processes. After the dissociation step, where dendritic endings are lost, adding glutamate as a supplement to the culture medium can be important. We believe that moderate addition of glutamate can promote the formation of dendritic endings in vitro with functional properties similar to those in vivo in SGNs. It is clear that afferent stimulation is necessary for neuron maturation, synaptic connection formation, and neuron survival. However, there are no data on which glutamate concentration in the culture medium is suitable for these purposes. Glutamate activity largely depends on concentration and duration of exposure. Vieira et al. mentioned adding 25 μM glutamate to the culture medium, as it may have a trophic effect [83]. Both ionotropic glutamate receptors (iGluRs) and mGluRs can enhance neuronal survival and protect neurons from damage. It is important to note that glutamate stimulation furthermore plays a modulatory role and affects the expression of glutamate receptors. This influences the functional response of SGNs.

Existing protocols for obtaining primary cell cultures vary across studies, which affects not only neuronal survival and development but also the reproducibility of methods using these primary cultures.

### 3.4. Functional Properties of the Glutamatergic System in Isolated SGNs

The study of the glutamatergic system in isolated SGNs is conducted using two main approaches: primary cell cultures and experiments on acutely isolated (non-cultured) cells. During the isolation of dissociated cell cultures, SGNs are subjected to mechanical and enzymatic dissociation. This process causes unavoidable damage to the afferent nerve endings and axons of SGNs. Immediately following dissociation, acutely isolated cells are rounded somata without neurites. Neurite outgrowth begins within a few hours. Neurons regain their bipolar shape, but some neurons may acquire multipolar and unipolar shapes [72]. Thus, in contrast to acutely isolated cells, neurons in primary culture have regrown dendrites. Therefore, neurons lacking dendrites or with regrown dendrites may exhibit different functional properties than those observed in vivo.

The data shown in Table 3 demonstrate a wide range of methods and recorded parameters applied in studies using glutamate receptor agonists and antagonists on primary SGCs. Research on the acutely isolated model was limited to patch-clamp techniques and functional calcium imaging employed to assess the responses to glutamate receptor agonists and antagonists. In studies using primary SGC cultures, researchers have assessed SGNs’ survival [69,70,75,79,80,81], neurite length [69,70], morphology [79,81], the expression levels of pro-apoptotic and anti-apoptotic factors [72,79,89], oxidative stress levels [79], and other parameters under glutamate excitotoxicity conditions. Consequently, when utilizing an isolated SGC model, it is important to consider that the processes of cell isolation and cultivation may lead to alterations in the expression, distribution, and subunit composition of glutamate receptors and ion channels [14].

Existing protocols for studying the glutamatergic system on primary cultures of SGCs predominantly utilize neonatal animals. However, the subunit composition of glutamate receptors changes depending on the developmental stage, both in the central nervous system and in the peripheral nervous system. These changes directly affect the nature of neuronal responses to glutamate [100]. IHC-SGN synapses undergo maturation, acquiring their final morphological and functional characteristics during the first two postnatal weeks, with the onset of hearing occurring at postnatal day 12 (P12) in mice [101,102]. Since the skull is not yet ossified in the early neonatal period [57], most existing protocols for primary SGC culture utilize newborns.

#### 3.4.1. Acutely Isolated Cells

Previous studies used acutely isolated cells either right after preparing the suspension or following brief culturing [18,29,30,36,37,76].

Using patch-clamp electrophysiology, Nakagawa et al. provided the first characterization of excitatory amino acid responses in isolated mammalian SGCs [29]. This study was among the first to show that postsynaptic responses in mammals are mediated primarily by non-NMDARs. The authors note the absence of dendrites in acutely isolated SGCs. Based on the approach of Feltz and Rasminsky in studying neurons of the lumbar spinal ganglia, Nakagawa proposed using the SGN soma and a short dendrite as a model of the primary afferent nerve terminal [103]. Nevertheless, the findings of this study were corroborated by in vivo investigations [15,18].

Harada et al. were the first to apply fluorescent calcium imaging to isolated SGCs specifically to study their response to L-glutamate [76]. Their critical finding was that the glutamate-induced increase in intracellular calcium ([Ca^2+^]_i_) was strictly dependent on the presence of neurites. Isolated SGCs without neurites did not respond to glutamate exposure. Therefore, the authors showed a dependence of SGNs’ function on morphology under in vitro conditions. Their work also provided critical evidence supporting the hypothesis that neurotransmission at the mammalian IHC-SGN synapse is mediated by non-NMDARs. These results were obtained indirectly in the presence of extracellular Mg^2+^, which blocks NMDARs.

Shimozono et al. first demonstrated on isolated SGCs that fast excitatory synaptic transmission at the mammalian IHC–SGN synapse is mediated by non-NMDARs, using specific agonists (kainate and quisqualate) [30]. They confirmed that [Ca^2+^]_i_ did not increase in response to NMDA and that the glutamate-induced response was not blocked by the NMDAR antagonist (APV). This provides further support for the findings of Harada et al. An important finding of this study was that non-NMDAR-mediated calcium influx is mediated primarily by voltage-gated calcium channels. In this study, patch-clamp electrophysiology and functional calcium imaging were applied in combination for the first time on isolated SGCs. This work contributed to a better understanding of glutamatergic signaling in SGNs. The authors employed an SGC isolation protocol akin to that of Nakagawa et al., who noted that neurons prepared this way lack processes. Notably, Shimozono et al. observed [Ca^2+^]_i_ rises in response to glutamate and non-NMDAR agonists. It is unclear if this conflicts with the report by Harada et al., who found that neurites are required for glutamate-induced calcium influx. Both studies did not specify pre-experimental incubation times. The cells in the Shimozono et al. study may have regrown processes before imaging.

In addition to existing in vivo studies, experiments on acutely isolated cells have provided further insights into the role of NMDARs in SGNs. Functional calcium imaging revealed the specific modulation of excitatory NMDAR responses by D-serine, rather than by glycine. Along with in vivo data, it has been suggested that D-serine is involved in noise-induced cochlear injuries, probably through potentiation of cochlear NMDARs [18]. It is important to note that the protocol of this study also does not specify the pre-experimental incubation time and does not describe cell morphology.

Cross-study comparison is difficult not only for survival data but also for functional measurements. When recording the same parameters, contradictory data may arise, which may be related to methodological features that are either specified or unspecified in the protocols. This complicates data interpretation. Shimozono et al. and Harada et al. used acutely isolated spiral ganglion cells derived from guinea pigs and performed functional calcium imaging using Fura-2. Both studies demonstrated an increase in intracellular Ca^2+^ concentrations ([Ca^2+^]_i_) in response to 100 μM glutamate application. Shimozono et al. reported an increase from 85 ± 9 nM to 445 ± 45 nM (mean ± SEM, *n* = 4–7), whereas Harada et al. observed a rise from 96 ± 17 nM to 241 ± 79 nM (mean ± SD, *n* = 21). The reported values for this response appear to vary across studies, although a direct comparison is not possible due to differences in sample size, methods of data presentation (SEM vs. SD), and experimental conditions. A comparison of the responses to glutamate and potassium reveals contrasting trends across studies. In the study by Shimozono et al., the response to glutamate exceeded that to potassium (445 vs. 349 nM), whereas the opposite pattern was observed in Harada et al. (241 vs. 504 nM). Several methodological factors may contribute to the observed variability. These include differences in Fura-2 loading concentration and animal weight. Specifically, Harada et al. used animals weighing 200–300 g, whereas Shimozono et al. used animals weighing 200–700 g. This difference likely reflects an age difference. Since the expression of receptors and ion channels changes during development, age may partially influence the observed responses. Additional uncertainty stems from the dissociation method. Shimozono et al. cite the work of Nakagawa et al., which describes two approaches: enzymatic dissociation (using collagenase) and mechanical dissociation (the “sucrose method”). Importantly, Akaike et al.demonstrated that enzymatic treatment reduces the sensitivity of NMDA receptors, whereas mechanical dissociation preserves it [104]. Since Shimozono et al. do not specify which method was used, it is impossible to assess the impact of this factor. Taken together, these differences indicate that, despite the apparent similarity of the experimental approaches, a quantitative comparison of response amplitudes across studies is not valid. This underscores the need for better standardization and more detailed descriptions of methods to ensure the reproducibility of results.

Wang et al. reported an EC_50_ value for glutamate of 45.8 μM, which decreased to 35.6 μM in the presence of D-serine [18]. At a glutamate concentration of 100 μM, intracellular calcium levels increased by 59.9 ± 4.9 nM (*n* = 59). It is important to note that this study was conducted on early postnatal rats (P1–P3), whereas the studies by Shimozono et al. and Harada et al. were conducted on adult guinea pigs. Given both interspecific differences and the marked dependence of glutamate receptor and calcium channel expression on the stage of development, these data are not directly comparable. In this case, the difference in response amplitude may reflect biological differences rather than methodological factors.

#### 3.4.2. Primary SGC Culture

An early study by Yamaguchi et al. utilized a long-term primary culture of chick embryo SGNs (5–14 days), in which neurons had the opportunity to regenerate their processes. They demonstrated that the excitatory postsynaptic potential (EPSP) is generated by NMDARs in these cells [55]. A key feature of this study was the utilization of non-mammalian neurons. Subsequent research has revealed distinct differences in neurotransmission between mammals and non-mammals. Historically, this work was the first to employ a primary culture of SGCs as a model system for investigating the glutamatergic system.

Subsequent studies have shown that the EPSP in mammals is mediated by AMPAR in the cochlea [15]. Chen et al., using a short-term SGC culture, showed NMDA-dependent regulation of the number of AMPARs on the surface of SGNs from newborn mice. This was one of the first pieces of evidence that NMDARs play a modulatory role in neurotransmission in the mammalian cochlea, unlike AMPARs. The researchers confirmed these results in in vivo experiments [36,37].

Using primary SGC culture, Peng et al. evaluated the role of metabotropic receptors. Metabotropic glutamate receptors are present in the cochlea, where they participate in auditory processing [41]. Peng et al. were among the first to demonstrate the functional role of mGluR1 in SGNs [13]. They combined in vitro approaches, including calcium imaging and patch-clamp, with in vivo experiments. The authors also revealed the protective effects of mGluR antagonists, suggesting the involvement of these receptors in the mechanisms of glutamate-induced excitotoxicity.

The study by Xiao et al. represents another example where functional calcium imaging was applied to primary cultures of SGC [77]. They demonstrated the neurotoxic effect of quinolinic acid (QA) on SGNs. This finding suggests a potential role of QA in the pathogenesis of SNHL associated with otitis media with effusion (OME). The authors demonstrated that quinolinic acid induces transient changes in intracellular calcium concentration in SGNs, which are mediated by NMDARs activation with functional calcium imaging. Quinolinic acid is known to play a role in various inflammatory diseases of the central nervous system. However, few studies have examined its neurotoxicity in the inner ear.

When studying primary SGC cultures, researchers use various methods, such as fluorescence and confocal microscopy, Western blot, RT-PCR, and viability assays (e.g., MTT). The application of this model has enabled key advances in understanding glutamate excitotoxicity in the inner ear. Lefebvre et al. were the first to establish an in vitro model of glutamate excitotoxicity in mammals using primary SGSs culture derived from both adult and neonatal rats [75]. Using primary cultures of SGCs from newborn rats, Ding and colleagues demonstrated the critical role of apoptosis-inducing factor (AIF) and calpain activation in glutamate-mediated damage and apoptosis of SGNs [78,89].

This model enables examination of otoprotective compound activity. In a study conducted by Sun et al. using primary cultures of neonatal mouse SGCs, a neuroprotective effect of natriuretic peptide was demonstrated in a model of glutamate excitotoxicity [81]. The number of neurons in the dish and the length of neurites were chosen as indicators to demonstrate the neuroprotective effect. Bai et al. demonstrated that Edaravone exerts a dose-dependent neuroprotective effect in primary SGC culture under glutamate excitotoxicity conditions, as evidenced by improved neuronal survival and reduced apoptotic markers [79]. Using this model, Li et al. demonstrated the neuroprotective properties of vancomycin [80].

A comparison of available data on SGC survival after glutamate exposure shows marked differences between laboratories. Lefebvre et al. reported less than 10% neuronal survival after exposure to 1 mM glutamate for 24 h. Bai et al. observed 30–32% survival using twice the concentration (2 mM), but with a much shorter exposure time of 10 min. Li et al. used the same concentration (2 mM) with a longer exposure of 23 h and found 43 ± 5% survival. Sun et al. reported approximately 50% neuronal loss at a much lower concentration of 100 μM over 48 h. Glutamate concentration ranged from 100 μM to 2 mM and exposure time from 10 min to 48 h. This makes it hard to assess the contribution of each factor separately. Second, the methods used to assess survival differed. Lefebvre et al. and Sun et al. counted neurons using immunocytochemistry. Li et al. used the MTT assay, which reflects cell metabolic activity. Bai et al. combined MTT with trypan blue staining. Their results are therefore not directly comparable. Third, animal age varied across studies, from embryonic day 18 to postnatal days P3 to P5. This may have affected receptor maturity and neuronal sensitivity to glutamate. Finally, studies differed in how data were reported: as percentage of control, absolute cell counts, or fold of normal. Different measures of variability were also used (SD and SEM), further limiting cross-study comparison. Despite the methodological variability, all studies confirm that the excitotoxicity phenomenon is reproducible in this model. The quantitative differences in survival rates primarily reflect methodological differences between studies.

These studies demonstrate that an in vitro primary SGC model can be used to simulate glutamate excitotoxicity and investigate the neuroprotective properties of compounds.

## 4. Discussion

The use of in vitro models is a widely adopted approach in auditory system research. The present review summarizes studies in which primary SGCs are employed as an experimental model to study glutamate transmission and to investigate the phenomenon of glutamate-induced excitotoxicity. Early studies of glutamatergic transmission in primary SGCs used chicken cells [29,55]. However, studies have shown a fundamental difference between birds and mammals. In chickens, NMDARs generate postsynaptic currents. In mammals, this role is performed by AMPAR [29]. Subsequent studies predominantly utilized mammalian models. Thus, when selecting an experimental animal model, it is necessary to take into account the presence of functional differences in the cells.

The process of cochlear isolation from the skull is associated with a high risk of mechanical damage to the soft tissues enclosed in the bony labyrinth. Cochlear isolation and inner ear tissue processing can be performed in experimental animals of varying ages [57]. However, neonatal mice or rats are the most common source of cochleae for creating in vitro and ex vivo models. At this age, the skull and cochlea are not yet ossified. This facilitates the isolation of the membranous labyrinth structures from the otic capsule [67]. In newborn mice, the removal of the bony cochlear capsule can be carried out without damaging the underlying tissues, including the modiolar region.

The functional properties of glutamatergic signaling change throughout postnatal development. This maturation is reflected in shifts in the expression profile and subunit composition of glutamate receptors, which ultimately shape the functional characteristics of SGNs. Research indicates that the period of greatest sensitivity to excitotoxicity caused by glutamate agonists is during the early stages of development. Specifically, this occurs in rats between postnatal days 9 and 12 [105]. Despite the technical difficulties associated with isolating SGCs from adult animals, such protocols have been described. However, due to the difficulty of isolating SGCs from the adult animal modiolus, protocols using neonatal animals predominate. It should be noted that neurons from rats up to P12 are taken from the pre-hearing period. Since the auditory system is not fully formed, the mechanisms of glutamate excitotoxicity in vitro may differ from those in adult animals [106]. This difference may be primarily due to variations in the subunit composition of glutamate receptors and, consequently, in their ionic permeability. Therefore, functional differences in SGCs at various developmental stages must be considered when investigating the role of glutamate and glutamate-induced excitotoxicity in isolated neurons.

The use of primary SGCs is associated with a number of limitations, primarily because the cell phenotype in vitro may differ from the phenotype in their native environment. Cell behavior in 2D monolayers in culture differs from that observed in vivo: morphology, receptor expression, and gene expression changes [92]. Thus, parameters of cell isolation and cultivation protocols may substantially influence experimental outcomes. Our analysis reveals significant heterogeneity in experimental protocols for isolating SGCs, especially regarding dissociation methods and culture conditions.

Enzymatic and mechanical dissociation leads to the loss of the natural cellular microenvironment and contacts with the extracellular matrix, which can trigger programmed cell death—anoikis [107]. Therefore, the interval between isolating cells from the modiolus and their attachment to a coating substrate should be minimized in time. Besides, mechanical and enzymatic dissociation alter gene expression profiles [73]. It has been observed that varying dissociation protocols result in different gene expression in cultured cells [74]. It remains unclear how much the dissociation process alters functional characteristics of SGNs. Therefore, it is important to investigate changes in the expression levels of key glutamate neurotransmission elements following isolation. This will minimize dissociation-induced artifacts.

It is known that histolytic enzymes can affect the properties of membrane receptors, reducing their binding capacity [108]. Trypsin has been shown to affect NMDA receptor-mediated responses in isolated hippocampal neurons, suggesting the presence of a trypsin-sensitive component in NMDA receptors [109]. Considering the modulatory role of NMDA receptors in SGNs demonstrated by Chen et al., it can be assumed that the response of SGNs to glutamate may be altered in experimental conditions. However, no data are currently available on the direct effects of dissociation enzymes on AMPA receptors mediating excitatory currents and excitotoxicity in SGNs. There is no convincing evidence that any of the histolytic enzymes affect the ligand-binding sites of glutamate receptors in SGNs. Additionally, receptor ionic permeability depends on the subunit composition of glutamate receptors expressed during neurite outgrowth after dissociation. This factor may also influence the experimental outcome.

Modiolus cell suspension culture contains neurons, satellite cells, Schwann cells, and fewer fibroblasts, astrocytes, and oligodendrocytes. Non-neuronal cells divide actively, proliferate and grow; accordingly, long-term culture of SGCs is difficult. This problem is especially severe in cultures of newborn animals. Using the mitotic inhibitor AraC was suggested as a method to improve the neuronal proportion in culture [68]. Thus, the loss of the natural intercellular environment, as well as the unrestricted growth of the non-neuronal component, requires improved approaches to culturing SGCs. This includes the use of specialized media, such as NBM, to enhance neuronal survival, as well as the use of supplements, particularly neurotrophic factors [83,90].

An alternative source of neurotrophic factors may be conditioned media derived from the culture of peripheral and central targets of SGNs, or co-culture systems [110,111]. Neurotrophic factors have been shown to influence the electrophysiological properties of neurons and modulate glutamate receptor expression [90,112]. Therefore, the use of preconditioned media, coculture of SGCs, or direct addition of BDNF and NT-3 to the culture medium is necessary not only for cell survival and formation of normal bipolar morphology, but also for maturation of glutamate receptors.

When modeling glutamate excitotoxicity in isolated SGCs, the composition of the culture environment critically determines neuronal responses to glutamate. Most studies on primary SGCs culture predominantly use serum-containing media (FBS). However, serum supplementation may itself confound excitotoxicity experiments. Cheng and Sontheimer reported that serum contains high levels of glutamate, which may render neurons cultured in serum-containing media less susceptible to acute excitotoxicity [113,114]. This reduced sensitivity may be attributed to chronic exposure to subtoxic glutamate doses present in serum, which could trigger changes in receptor expression, subunit composition, or receptor desensitization [115]. Additionally, such exposure may promote selective survival of neuronal populations inherently resistant to excitotoxic injury [113,116]. Additionally, serum contains substances with direct neuroprotective actions against glutamate excitotoxicity [117,118]. On the other side, serum-containing media may also potentiate excitotoxicity [114,119]. Such conflicting results likely reflect the undefined nature of serum, whose composition varies between lots and can significantly affect experimental reproducibility. No comparative studies have been done on how culturing SGCs in serum-free versus serum-containing media affects glutamate excitotoxicity in SGCs.

Serum-free supplements are optimized to maximize the survival of cultured neurons. The most widely used supplements for serum-free neuronal culture are B27 and N2. They contain numerous ingredients, many of which act as enzymatic or non-enzymatic antioxidants [120]. Antioxidants protect cultured neurons from oxidative stress induced by the production of free radicals, which is the direct result of glutamate excitotoxicity. These supplements can shift dose–response curves and reduce glutamate toxicity [121,122].

Based on the above, glutamate concentrations used to model excitotoxicity vary widely across studies. For functional approaches, however, the range of concentrations used across studies is considerably narrower. In several studies, responses are characterized as dose–response curves. For calcium imaging, 100 μM appears to be the most widely adopted concentration. For patch-clamp recordings in mammalian SGNs, concentrations in the range of 100–300 μM have been predominantly employed. These findings may prove useful for researchers seeking to reproduce and validate primary SGN culture models in their laboratories.

Alternative models for studying glutamatergic transmission include cochlear explants. The advantage of these models is the presence of the intact IHC/SGN type I synapses, which allows the study of glutamate-dependent mechanisms of cochlear synaptopathy. In contrast, this cannot be done using isolated SGCs. However, unlike whole cochlear explants, 2D cell cultures are more convenient for single-cell methods such as functional calcium imaging and patch-clamp. There are no standardized approaches to obtaining or using a primary SGC model for in vitro studying the glutamatergic system. The appropriate model should be selected based on the specific research question.

The present review summarizes the accumulated experience from previous studies in the field. The resulting analysis may be useful to researchers planning to work with the model.

## 5. Conclusions

The isolated SGC model continues to be actively used in experimental otolaryngology. The properties of neurons under in vitro conditions clearly differ from those in vivo. This limitation is inherent to any in vitro system in principle. This complicates the interpretation of experimental data because the cell phenotype, including receptor expression, is inevitably altered by the cell isolation methods and artificial culture conditions. The protocols for obtaining and culturing primary SGCs demonstrate heterogeneity in existing studies, making it difficult to compare results between different studies. Despite this, there is already an understanding of how dissociation and cultural conditions affect the structure and function of primary SGNs.

Besides some limitations, the primary SGC model in vitro has made significant contributions to understanding the glutamatergic system and excitotoxicity mechanisms in auditory neurons. Acutely isolated SGCs can be used to determine signal transduction and receptor function. Cultured cells are better suited for studying calcium dysregulation and the mechanisms of glutamate excitotoxicity or otoprotection. The number of neurons in a Petri dish and also the length of neurites can serve as good indicators of the neuroprotective effect under conditions of glutamate excitotoxicity. Thus, although the lack of in vivo microenvironmental cues significantly reduces the physiological relevance of in vitro studies, the ability to manipulate SGCs outside the body represents an important advantage for understanding the specific mechanisms of their death and survival.

To improve reproducibility in studies of the glutamatergic system of SGNs in vitro, future works should report key primary culture parameters in detail. These include SGC density in culture vessels, the neuronal/non-neuronal ratio, pre-exposure cultivation time, and maximum incubation period. Functional assays and survival tests should be accompanied by detailed morphological characterization of neurites. Any protocol optimization should include a concentration–response curve for effects mediated by glutamate receptor agonists. The use of conditioned media from peripheral targets (organ of Corti cultures) or central targets (astroglial cultures), as well as co-culture systems, may provide more physiological conditions for primary SGCs in vitro. Suppression of active non-neuronal proliferation in SGC cultures from neonatal animals can be achieved by adding cell division inhibitors (e.g., AraC). Comparing serum-free conditions based on Neurobasal/B27 media with conventional serum-containing media based on DMEM/FBS would help identify the most reproducible conditions for studying glutamate receptor expression, function, and excitotoxicity in vitro. For calcium imaging and patch-clamp experiments, researchers should refer to the glutamate concentration ranges established in published studies.

## Figures and Tables

**Table 2:** Key methodological parameters of primary SGC cultures used in glutamate signaling research. Note: Not reported data are presented by the symbol “NA”.

Study	Animal Species and Age	Long-Term/Short-Term Culture	Coating Substrate	Medium and Supplements	Pre-Exposure Cultivation Time/ Maximum Incubation Period
Yamaguchi and Ohmori, 1990 [55]	Chick embryo (16–19 embryonic days)	Long-term	Collagen Poly-D-lysine	**DMEM+F12** calf serum	5–14 days/5–14 days
		Short-term	Poly-D-lysine/ concanavalin A	**DMEM+F12** calf serum	<1 day/<1 day
Lefebvre et al., 1991 [75]	Rats (P5)	Long-term	Poly-ornithine; Laminin;	**DMEM+** N1 cocktail: bovine insulin, progesterone, putrescine, transferrin, selenium	5 days/6 days
Lefebvre et al., 1991 [75]	Adult rats	Long-term	Poly-ornithine; Laminin; Astrocyte-conditioned medium	**DMEM+** N1 cocktail: bovine insulin, progesterone, putrescine, transferrin, selenium	3 days/4 days
Peng et al., 2004 [13]	Mice (P0, adult)	Long-term	NA	**DMEM+** FBS penicillin streptomycin	2 days/2 days and more
Zhai et al., 2004 [70]	Mice (P3)	Long-term	Rat-tail collagen	Not fully specified	24 h/14 days
Chen et al., 2007 [36]	Mice (P6–P8)	Short-term	Gelatin	**DMEM+F12** FBS, horse serum, NT-3, BDNF, penicillin and streptomycin	15–18 h/15–18 h + ~1 h
Chen et al., 2009 [37]	Mice (P3–P5)	Short-term	Poly-L-ornithine	**DMEM+F12** FBS, horse serum, NT-3, BDNF, B-27 supplement, penicillin and streptomycin	15–18 h/15–18 h + ~24 h
Xiao et al., 2010 [77]	Rats (P3–P7)	Long-term	NA	**NBM** B27 AraC	72 h/96 h
Ding et al., 2015 [78]	Rats (P1)	Long-term	NA	**DMEM** B27, BDNF, penicillin	24 h/72 h
Ding et al., 2015 [89]	Rats (P0–P3)	Long-term culture	NA	**DMEM** B27, BDNF, penicillin	NA
Bai et al., 2016 [79]	Rats (<P5)	Short-term culture	Poly-L-lysine	**DMEM** FBS	NA
Li et al., 2018 [80]	Rats (embryonic day-18)	Short-term culture	Poly-L-lysine	**DMEM** FBS	NA
Sun et al., 2021 [81]	Rats (P3)	Long-term culture	Poly-L-lysine	**DMEM** FBS N2 NT-3	4 h/5 days

**Table 3:** Summary of Experimental Studies on Glutamate Receptor Agonists and Antagonists in Primary SGCs. Note: Not reported data are presented by the symbol “NA”.

Study	Animal Species and Age	Methods (Application)	Glu Receptor Agonists (Concentration)	Glu Receptors Antagonists (Concentration)
Yamaguchi and Ohmori, 1990 [55]	Chick embryo (16–19 embryonic days)	Patch-clamp (Ionic currents); Fluorescent microscopy (Cell morphology)	Glu (30 μM) KA (100 μM) Asp (100 μM) NMDA (100 μM)	APV (100 μM)
Lefebvre et al., 1991 [75]	Rats(P5); Adult rats	Immunohistochemical staining (Survival, cell morphology)	Rats (P5): Glu 10^−8^–10^−3^ M NMDA 10^−8^–10^−3^ M Kainic acid 10^−8^–10^−3^ M Quisqualic acid 10^−8^–10^−3^ M Adult rats: Glu (10^−4^ M) NMDA (10^−4^ M) Kainic acid (10^−4^ M) Quisqualic acid (10^−4^ M)	Rats (P5): DAP-5 (10^−9^–10^−3^ M) Kyn (10^−9^–10^−6^ M) Adult rats: DAP-5 (10^−4^ M) Kyn (10^−4^ M)
Nakagawa et al., 1991 [29]	Adult guinea pigs; Chickens from 2 to 5 wk post-hatch	Patch-clamp (Ionic currents); Phase-contrast microscopy (Cell morphology)	Adult guinea pigs: Glu (3 × 10^−6^–10^−2^ M) QA (3 × 10^−7^–3 × 10^−4^ M) KA (3 × 10^−6^–3 × 10^−4^ M) Asp (3 × 10^−3^ M) NMDA (3 × 10^−3^ M) Chicken Glu (3 × 10^−4^ M) QA (3 × 10^−5^ M) KA (10^−4^ M) Asp (10^−3^ M) NMDA (3 × 10^−3^ M)	Adult guinea pigs: CNQX (10^−8^–10^−5^ M); DNQX (10^−8^–10^−5^ M); diCl-HQC (10^−7^–10^−4^ M); Kyn (3 × 10^−6^–3 × 10^−3^ M); APV (3 × 10^−5^ M) Chicken APV (3 × 10^−5^ M)
Harada et al., 1994 [76]	Adult guinea pigs	Calcium imaging (Intracellular calcium [Ca^2+^]_i_ concentration); Phase-contrast microscopy (Cell morphology)	Glu (100 μM)	NA
Shimozono et al., 1995 [30]	Adult guinea pigs	Patch-clamp (Ionic currents); Calcium imaging (Intracellular calcium [Ca^2+^]_i_ concentration)	Glu (10, 50, 100 μM) KA (100 μM); NMDA (100 μM); QA (100 μM)	APV (NA);
Peng et al., 2004 [13]	Mice (P0, adult)	Patch-clamp (Ionic currents) Calcium imaging (Intracellular calcium [Ca^2+^]_i_ concentration)	KA (100 μM); Glu (300 μM) DHPG (100 μM); ACPD (100 μM)	AIDA (300 μM) DNQX (200 μM)
Zhai et al., 2004 [70]	Mice (P3)	Histochemical staining (Survival, neurite length)	Glu (20 mM)	NA
Chen et al., 2007 [36]	Mice (P6–P8)	Immunofluorescence staining (surface GluR2 expression, cell viability)	Glu (20 μM) AMPA (20 μM) NMDA (20 μM)	DNQX (20 μM) APV (50 μM)
Chen et al., 2009 [37]	Mice (P3–P5)	Immunofluorescence staining (surface GluR2 expression, cell viability)	AMPA (20 μM) NMDA (20 μM) AMPA (300 μM) NMDA (300 μM)	NA
Xiao et al., 2010 [77]	Rats (P3–P7)	Fluorescent microscopy (morphology, apoptosis) Laser confocal microscopy (Intracellular calcium [Ca^2+^]_i_ concentration)	Quinolinic acid (100; 1000 μM/L)	MK-801 (20 μM/L)
Ding et al., 2015 [78]	Rats (P1)	Stereoscopic microscopy (Cell morphology) Immunofluorescence staining (AIF distribution) TUNEL assay (Apoptosis) Western Blot & RT-PCR (AIF, calpain, caspase-3 expression)	Glu (20 μM)	NA
Ding et al., 2015 [89]	Rats (P0–P3)	Immunofluorescence staining (AIF distribution) RT-PCR (AIF, calpain, caspase-3 expression)	Glu (10 mM, 20 mM, 40 mM)	NA
Bai et al., 2016 [79]	Rats (P < 5)	MTT assay & Trypan blue staining (Cell viability) Trypan blue staining (Cell viability) Ho.33342 and Propidium iodide double staining (Apoptosis and necrosis) Spectrophotometry (GSH content, SOD activity, MDA level) Western Blot (AKT, p-AKT, Bax, Bcl-2 expression levels)	Glu (2 mM)	NA
Li et al., 2018 [80]	Rat embryo (18 embryonic days)	MTT assay (Cell viability) Ho.33342 and Propidium iodide double staining (Apoptosis and necrosis)	Glu (2 mM)	NA
Sun et al., 2021 [81]	Rats (P3)	Immunofluorescence staining (Neuronal number and neurite length)	Glu (100 μM)	NA
Wang et al., 2021 [18]	Rats (P1–P4)	Calcium imaging (Intracellular calcium [Ca^2+^]_i_ concentration)	AMPA (100 μM) Glu (100 μM) NMDA (100 μM) Glu (100 μM) + D-serine (100 μM)	7-CKA (50 μM) D-APV (50 μM) GYKI 53784 (40 μM)

## Data Availability

This review only presents data that have been previously published. No new data were generated.

## Abbreviations

The following abbreviations are used in this manuscript:

7-CKA	7-chlorokynurenic acid
ACPD	(1S,3R)-aminocyclopentane-1,3-dicarboxylic acid
AIDA	(RS)-1-aminoindan-1,5-dicarboxylic acid
AIF	Apoptosis-inducing factor
AMPA	α-amino-3-hydroxy-5-methyl-4-isoxazolepropionic acid
AMPARs	α-amino-3-hydroxy-5-methyl-4-isoxazolepropionic acid receptors
Asp	Aspartic acid
AraC	Cytosine β-D-arabinofuranoside
BDNF	Brain-derived neurotrophic factor
CI-AMPARs	Calcium-impermeable AMPARs
CNQX	6-cyano-7-nitroquinoxaline-2,3-dione
CP-AMPARs	Calcium-permeable AMPARs
DAP-5	2-amino-5-phosphonovalerate
DHPG	(S)-3,5-Dihydroxyphenylglycine
diCl-HQC	6,7-dichloro-3-hydroxy-2-quinoxalinecarboxylic acid
DNQX	6,7-dinitroquinoxaline-2,3-dione
EPSP	Excitatory postsynaptic potential
Glu	Glutamate
GRM7	Metabotropic glutamate receptor 7
GYKI 53784	1-(4-aminophenyl)-4-methyl-7, 8-methylenedioxy-4,5-dihydro-3-methylcarbamoyl-2,3-benzodiazepine
iGluRs	Ionotropic glutamate receptors
KA	Kainic acid
KARs	Kainic acid receptors
Kyn	Kynuranic acid
mGluRs	Metabotropic glutamate receptors
MK-801	Dizocilpine
NBM	Neurobasal medium
NMDA	N-methyl-D-aspartate
NMDARs	N-methyl-D-aspartate receptors
NSAIDs	Nonsteroidal anti-inflammatory drug
NT-4/5	Neurotrophin-4/5
NT3	Neurotrophin-3
OME	Otitis media with effusion (OME)
QA	Quisqualic acid
ROS	Reactive oxygen species
SGCs	Spiral ganglion cells
SGNs	Spiral ganglion neurons

## References

[1] Chadha S., Kamenov K., Cieza A. (2021). The world report on hearing, 2021. Bull. World Health Organ..

[2] Alford R.L., Arnos K.S., Fox M., Lin J.W., Palmer C.G., Pandya A., Rehm H.L., Robin N.H., Scott D.A., Yoshinaga-Itano C. (2014). American college of medical genetics and genomics guideline for the clinical evaluation and etiologic diagnosis of hearing loss. Genet. Med..

[3] Natarajan N., Batts S., Stankovic K.M. (2023). Noise-Induced Hearing Loss. J. Clin. Med..

[4] Pasdelou M.P., Byelyayeva L., Malmström S., Pucheu S., Peytavy M., Laullier H., Hodges D.B., Tzafriri A.R., Naert G. (2024). Ototoxicity: A high risk to auditory function that needs to be monitored in drug development. Front. Mol. Neurosci..

[5] Yang W., Zhao X., Chai R., Fan J. (2023). Progress on mechanisms of age-related hearing loss. Front. Neurosci..

[6] Bing X., Liu C., Cao X., Li C., Gao X., Zhu F., Wu X., Guo N., Hu H., Xia M. (2025). Development of the inner ear and regeneration of hair cells after hearing impairment. Fundam. Res..

[7] Blankenship C., Zhang F., Keith R. (2016). Behavioral measures of temporal processing and speech perception in cochlear implant users. J. Am. Acad. Audiol..

[8] Lesica N.A. (2018). Why Do Hearing Aids Fail to Restore Normal Auditory Perception?. Trends Neurosci..

[9] Barclay M., Ryan A.F., Housley G.D. (2011). Type I vs type II spiral ganglion neurons exhibit differential survival and neuritogenesis during cochlear development. Neural Dev..

[10] Nayagam B.A., Muniak M.A., Ryugo D.K. (2011). The spiral ganglion: Connecting the peripheral and central auditory systems. Hear. Res..

[11] Coate T.M., Kelley M.W. (2013). Making connections in the inner ear: Recent insights into the development of spiral ganglion neurons and their connectivity with sensory hair cells. Semin. Cell Dev. Biol..

[12] Niedzielski A.S., Wenthold R.J. (1995). Expression of AMPA, Kainate, and NMDA Receptor Subunits in Cochlear and Vestibular Ganglia. J. Neurosci..

[13] Peng B.G., Li Q.X., Ren T.Y., Ahmad S., Chen S.P., Chen P., Lin X. (2004). Group I metabotropic glutamate receptors in spiral ganglion neurons contribute to excitatory neurotransmissions in the cochlea. Neuroscience.

[14] Reijntjes D.O.J., Pyott S.J. (2016). The afferent signaling complex: Regulation of type I spiral ganglion neuron responses in the auditory periphery. Hear. Res..

[15] Ruel J., Chen C., Pujol R., Bobbin R.P., Puel J.L. (1999). AMPA-preferring glutamate receptors in cochlear physiology of adult guinea-pig. J. Physiol..

[16] Glowatzki E., Fuchs P.A. (2002). Transmitter release at the hair cell ribbon synapse. Nat. Neurosci..

[17] Eybalin M., Caicedo A., Renard N., Ruel J., Puel J.L. (2004). Transient Ca^2+^-permeable AMPA receptors in postnatal rat primary auditory neurons. Eur. J. Neurosci..

[18] Wang J., Serratrice N., Lee C.J., François F., Sweedler J.V., Puel J.L., Mothet J.P., Ruel J. (2021). Physiopathological Relevance of D-Serine in the Mammalian Cochlea. Front. Cell. Neurosci..

[19] Paoletti P., Bellone C., Zhou Q. (2013). NMDA receptor subunit diversity: Impact on receptor properties, synaptic plasticity and disease. Nat. Rev. Neurosci..

[20] Sebe J.Y., Cho S., Sheets L., Rutherford M.A., von Gersdorff H., Raible D.W. (2017). Ca^2+^-permeable AMPARs mediate glutamatergic transmission and excitotoxic damage at the hair cell ribbon synapse. J. Neurosci..

[21] Hu N., Rutherford M.A., Green S.H. (2020). Protection of cochlear synapses from noise-induced excitotoxic trauma by blockade of Ca^2+^-permeable AMPA receptors. Proc. Natl. Acad. Sci. USA.

[22] Walia A., Lee C., Hartsock J., Goodman S.S., Dolle R., Salt A.N., Lichtenhan J.T., Rutherford M.A. (2021). Reducing Auditory Nerve Excitability by Acute Antagonism of Ca^2+^-Permeable AMPA Receptors. Front. Synaptic Neurosci..

[23] Steinbach S., Lutz J. (2007). Glutamate induces apoptosis in cultured spiral ganglion explants. Biochem. Biophys. Res. Commun..

[24] Lewerenz J., Maher P. (2015). Chronic glutamate toxicity in neurodegenerative diseases-What is the evidence?. Front. Neurosci..

[25] Pinelis V., Krasilnikova I., Bakaeva Z., Surin A., Boyarkin D., Fisenko A., Krasilnikova O., Pomytkin I. (2022). Insulin Diminishes Superoxide Increase in Cytosol and Mitochondria of Cultured Cortical Neurons Treated with Toxic Glutamate. Int. J. Mol. Sci..

[26] Bakaeva Z., Goncharov M., Frolov F., Krasilnikova I., Sorokina E., Zgodova A., Smolyarchuk E., Zavadskiy S., Andreeva L., Myasoedov N. (2024). Regulatory Peptide Pro-Gly-Pro Accelerates Neuroregeneration of Primary Neuroglial Culture after Mechanical Injury in Scratch Test. Int. J. Mol. Sci..

[27] Shedenkova M., Gurianova A., Krasilnikova I., Sudina A., Karpulevich E., Maksimov Y., Samburova M., Guguchkin E., Nefedova Z., Babenko V. (2025). Extracellular Vesicles from iPSC-Derived Glial Progenitor Cells Prevent Glutamate-Induced Excitotoxicity by Stabilising Calcium Oscillations and Mitochondrial Depolarisation. Cells.

[28] Yamakura T., Shimoji K. (1999). Subunit-and site-specific pharmacology of the NMDA receptor channel. Prog. Neurobiol..

[29] Nakagawa T., Komune S., Uemura T., Akaike N. (1991). Excitatory Amino Acid Response in Isolated Spiral Ganglion Cells of Guinea Pig Cochlea. J. Neurophysiol..

[30] Shimozono M., Tono T., Morimitsu T., Nakagawa T., Komune S. (1995). Measurement of intracellular free Ca concentration in guinea pig spiral ganglion cells. NeuroReport.

[31] Hong J., Chen Y., Zhang Y., Li J., Ren L., Yang L., Shi L., Li A., Zhang T., Li H. (2018). N-Methyl-D-Aspartate Receptors Involvement in the Gentamicin-Induced Hearing Loss and Pathological Changes of Ribbon Synapse in the Mouse Cochlear Inner Hair Cells. Neural Plast..

[32] Guitton M.J., Caston J., Ruel J., Johnson R.M., Pujol R., Puel J.-L. (2003). Salicylate Induces Tinnitus through Activation of Cochlear NMDA Receptors. J. Neurosci..

[33] Ruel J., Chabbert C., Nouvian R., Bendris R., Eybalin M., Leger C.L., Bourien J., Mersel M., Puel J.L. (2008). Salicylate enables cochlear arachidonic-acid-sensitive NMDA receptor responses. J. Neurosci..

[34] Bing D., Lee S.C., Campanelli D., Xiong H., Matsumoto M., Panford-Walsh R., Wolpert S., Praetorius M., Zimmermann U., Chu H. (2015). Cochlear NMDA receptors as a therapeutic target of noise-induced tinnitus. Cell. Physiol. Biochem..

[35] Tang X., Zhu X., Ding B., Walton J.P., Frisina R.D., Su J. (2014). Age-related hearing loss: GABA, nicotinic acetylcholine and NMDA receptor expression changes in spiral ganglion neurons of the mouse. Neuroscience.

[36] Chen Z., Kujawa S.G., Sewell W.F. (2007). Auditory sensitivity regulation via rapid changes in expression of surface AMPA receptors. Nat. Neurosci..

[37] Chen Z., Peppi M., Kujawa S.G., Sewell W.F. (2009). Regulated expression of surface AMPA receptors reduces excitotoxicity in auditory neurons. J. Neurophysiol..

[38] Sanchez J.T., Ghelani S., Otto-Meyer S. (2015). From development to disease: Diverse functions of NMDA-type glutamate receptors in the lower auditory pathway. Neuroscience.

[39] Zhang-Hooks Y.X., Agarwal A., Mishina M., Bergles D.E. (2016). NMDA Receptors Enhance Spontaneous Activity and Promote Neuronal Survival in the Developing Cochlea. Neuron.

[40] Klotz L., Wendler O., Frischknecht R., Shigemoto R., Schulze H., Enz R. (2019). Localization of group II and III metabotropic glutamate receptors at pre- and postsynaptic sites of inner hair cell ribbon synapses. FASEB J..

[41] Lu Y. (2014). Metabotropic glutamate receptors in auditory processing. Neuroscience.

[42] Kleinlogel S., Oestreicher E., Arnold T., Ehrenberger K., Felix D. (1999). Metabotropic glutamate receptors group I are involved in cochlear neurotransmission. NeuroReport.

[43] Friedman R.A., Van Laer L., Huentelman M.J., Sheth S.S., Van Eyken E., Corneveaux J.J., Tembe W.D., Halperin R.F., Thorburn A.Q., Thys S. (2009). GRM7 variants confer susceptibility to age-related hearing impairment. Hum. Mol. Genet..

[44] Klotz L., Enz R. (2021). MGluR7 is a presynaptic metabotropic glutamate receptor at ribbon synapses of inner hair cells. FASEB J..

[45] Ma X., Guo J., Fu Y., Shen C., Jiang P., Zhang Y., Zhang L., Yu Y., Fan J., Chai R. (2022). G protein-coupled receptors in cochlea: Potential therapeutic targets for hearing loss. Front. Mol. Neurosci..

[46] Liberman M.C., Kujawa S.G. (2017). Cochlear synaptopathy in acquired sensorineural hearing loss: Manifestations and mechanisms. Hear. Res..

[47] Ma K., Zhang A., She X., Yang H., Wang K., Zhu Y., Gao X., Cui B. (2021). Disruption of Glutamate Release and Uptake-Related Protein Expression After Noise-Induced Synaptopathy in the Cochlea. Front. Cell Dev. Biol..

[48] Moverman D.J., Liberman L.D., Kraemer S., Corfas G., Liberman M.C. (2023). Ultrastructure of noise-induced cochlear synaptopathy. Sci. Rep..

[49] Wang M., Xu L., Han Y., Wang X., Chen F., Lu J., Wang H., Liu W. (2022). Regulation of Spiral Ganglion Neuron Regeneration as a Therapeutic Strategy in Sensorineural Hearing Loss. Front. Mol. Neurosci..

[50] Safina D.R., Surin A.M., Pinelis V.G., Kostrov S.V. (2015). Effect of neurotrophin-3 precursor on glutamate-induced calcium homeostasis deregulation in rat cerebellum granule cells. J. Neurosci. Res..

[51] Schonkeren S.L., Küthe T.T., Idris M., Bon-Frauches A.C., Boesmans W., Melotte V. (2022). The gut brain in a dish: Murine primary enteric nervous system cell cultures. Neurogastroenterol. Motil..

[52] Vargova I., Kriska J., Kwok J.C.F., Fawcett J.W., Jendelova P. (2022). Long-Term Cultures of Spinal Cord Interneurons. Front. Cell. Neurosci..

[53] Weidinger A., Milivojev N., Hosmann A., Duvigneau J.C., Szabo C., Törö G., Rauter L., Vaglio-Garro A., Mkrtchyan G.V., Trofimova L. (2023). Oxoglutarate dehydrogenase complex controls glutamate-mediated neuronal death. Redox Biol..

[54] Cramer T.M.L., Tyagarajan S.K. (2024). Protocol for the culturing of primary hippocampal mouse neurons for functional in vitro studies. STAR Protoc..

[55] Yamaguchi K., Ohmori H. (1990). Voltage-gated and chemically gated ionic channels in the cultured cochlear ganglion neurone of the chick. J. Physiol..

[56] Sun G., Liu W., Fan Z., Zhang D., Han Y., Xu L., Qi J., Zhang S., Gao B.T., Bai X. (2016). The Three-Dimensional Culture System with Matrigel and Neurotrophic Factors Preserves the Structure and Function of Spiral Ganglion Neuron in Vitro. Neural Plast..

[57] Meas S.J., Nishimura K., Scheibinger M., Dabdoub A. (2018). In vitro methods to cultivate spiral ganglion cells, and purification of cellular subtypes for induced neuronal reprogramming. Front. Neurosci..

[58] Szabo Z., Harasztosi C., Szucs G., Sziklai I., Rusznak Z. (2003). A detailed procedure and dissection guide for the isolation of spiral ganglion cells of the guinea pig for electrophysiological experiments. Brain Res. Brain Res. Protoc..

[59] Parker M., Brugeaud A., Edge A.S.B. (2010). Primary culture and plasmid electroporation of the murine organ of Corti. J. Vis. Exp..

[60] Grant L., Yi E., Goutman J.D., Glowatzki E. (2011). Postsynaptic recordings at afferent dendrites contacting cochlear inner hair cells: Monitoring multivesicular release at a ribbon synapse. J. Vis. Exp..

[61] Montgomery S.C., Cox B.C. (2016). Whole mount dissection and immunofluorescence of the adult mouse cochlea. J. Vis. Exp..

[62] May-Simera H. (2016). Evaluation of planar-cell-polarity phenotypes in ciliopathy mouse mutant cochlea. J. Vis. Exp..

[63] Landegger L.D., Dilwali S., Stankovic K.M. (2017). Neonatal murine cochlear explant technique as an in vitro screening tool in hearing research. J. Vis. Exp..

[64] Sakamoto A., Kuroda Y., Kanzaki S., Matsuo K. (2017). Dissection of the auditory bulla in postnatal mice: Isolation of the middle ear bones and histological analysis. J. Vis. Exp..

[65] Fang Q.-J., Wu F., Chai R., Sha S.-H. (2019). Cochlear Surface Preparation in the Adult Mouse. J. Vis. Exp..

[66] Ballesteros A., Swartz K.J. (2020). Dextran Labeling and Uptake in Live and Functional Murine Cochlear Hair Cells. J. Vis. Exp..

[67] Hahnewald S., Roccio M., Tscherter A., Streit J., Ambett R., Senn P. (2016). Spiral ganglion neuron explant culture and electrophysiology on multi electrode arrays. J. Vis. Exp..

[68] Schwieger J., Esser K.H., Lenarz T., Scheper V. (2016). Establishment of a long-term spiral ganglion neuron culture with reduced glial cell number: Effects of AraC on cell composition and neurons. J. Neurosci. Methods.

[69] Du H., Zhou X., Shi L., Xia M., Wang Y., Guo N., Hu H., Zhang P., Yang H., Zhu F. (2022). Shikonin Attenuates Cochlear Spiral Ganglion Neuron Degeneration by Activating Nrf2-ARE Signaling Pathway. Front. Mol. Neurosci..

[70] Zhai S.Q., Wang D.J., Wang J.L., Han D.Y., Yang W.Y. (2004). Basic fibroblast growth factor protects auditory neurons and hair cells from glutamate neurotoxicity and noise exposure. Acta Otolaryngol..

[71] Anacker A., Esser K.H., Lenarz T., Paasche G. (2022). Purification of Fibroblasts From the Spiral Ganglion. Front. Neurol..

[72] Lie M., Grover M., Whitlon D.S. (2010). Accelerated neurite growth from spiral ganglion neurons exposed to the Rho kinase inhibitor H-1152. Neuroscience.

[73] Machado L., Relaix F., Mourikis P. (2021). Stress relief: Emerging methods to mitigate dissociation-induced artefacts. Trends Cell Biol..

[74] Uniken Venema W.T.C., Ramírez-Sánchez A.D., Bigaeva E., Withoff S., Jonkers I., McIntyre R.E., Ghouraba M., Raine T., Weersma R.K., Franke L. (2022). Gut mucosa dissociation protocols influence cell type proportions and single-cell gene expression levels. Sci. Rep..

[75] Lefebvre P.P., Weber T., Leprince P., Rigo J.-M., Delr6e P., Rogister B., Moonen G. (1991). Kainate and NMDA toxicity for cultured developing and adult rat spiral ganglion neurons: Further evidence for a glutamatergic excitatory neurotransmission at the inner hair cell synapse. Brain Res..

[76] Harada N., Han D.-Y., Komeda M., Yamashita T. (1994). Glutamate-induced Intracellular Ca^2+^ Elevation in Isolated Spiral Ganglion Cells of the Guinea Pig Cochlea. Acta Otolaryngol..

[77] Xiao H., Yang C., He Y., Zheng N. (2010). Neurotoxicity of quinolinic acid to spiral ganglion cells in rats. J. Huazhong Univ. Sci. Technol.-Med. Sci..

[78] Ding Z.J., Chen X., Tang X.X., Wang X., Song Y.L., Chen X.D., Mi W.J., Wang J., Lin Y., Chen F.Q. (2015). Calpain inhibitor PD150606 attenuates glutamate induced spiral ganglion neuron apoptosis through apoptosis inducing factor pathway in vitro. PLoS ONE.

[79] Bai X., Zhang C., Chen A., Liu W., Li J., Sun Q., Wang H. (2016). Protective Effect of Edaravone on Glutamate-Induced Neurotoxicity in Spiral Ganglion Neurons. Neural Plast..

[80] Li Y., Zou Q., Zhang J. (2018). Vincamine exerts protective effect on spiral ganglion neurons in endolymphatic hydrops guinea pig models. Am. J. Transl. Res..

[81] Sun F., Zhou K., Tian K.Y., Zhang X.Y., Liu W., Wang J., Zhong C.P., Qiu J.H., Zha D.J. (2021). Atrial Natriuretic Peptide Promotes Neurite Outgrowth and Survival of Cochlear Spiral Ganglion Neurons in vitro Through NPR-A/cGMP/PKG Signaling. Front. Cell Dev. Biol..

[82] Zuo W.Q., Hu Y.J., Yang Y., Zhao X.Y., Zhang Y.Y., Kong W., Kong W.J. (2015). Sensitivity of spiral ganglion neurons to damage caused by mobile phone electromagnetic radiation will increase in lipopolysaccharide-induced inflammation in vitro model. J. Neuroinflammation.

[83] Vieira M., Christensen B.L., Wheeler B.C., Feng A.S., Kollmar R. (2007). Survival and stimulation of neurite outgrowth in a serum-free culture of spiral ganglion neurons from adult mice. Hear. Res..

[84] Whitlon D.S., Ketels K.V., Coulson M.T., Williams T., Grover M., Edpao W., Richter C.P. (2006). Survival and morphology of auditory neurons in dissociated cultures of newborn mouse spiral ganglion. Neuroscience.

[85] Kaiser O., Paasche G., Stöver T., Ernst S., Lenarz T., Kral A., Warnecke A. (2013). TGF-beta superfamily member activin A acts with BDNF and erythropoietin to improve survival of spiral ganglion neurons in vitro. Neuropharmacology.

[86] Schwieger J., Warnecke A., Lenarz T., Esser K.H., Scheper V., Forsythe J. (2015). Neuronal survival, morphology and outgrowth of spiral ganglion neurons using a defined growth factor combination. PLoS ONE.

[87] Hegarty J.L., Kay A.R., Green S.H. (1997). Trophic Support of Cultured Spiral Ganglion Neurons by Depolarization Exceeds and Is Additive with that by Neurotrophins or cAMP and Requires Elevation of [Ca^2+^]_i_ within a Set Range. J. Neurosci..

[88] Han D.Y., Yamashita T., Harada N., Kumazawa T. (1993). Calcium mobilization in isolated cochlear spiral ganglion cells of the Guinea pig. Acta Otolaryngol..

[89] Ding Z.J., Chen X., Tang X.X., Wang X., Song Y.L., Chen X.D., Wang J., Wang R.F., Mi W.J., Chen F.Q. (2015). Apoptosis-inducing factor and calpain upregulation in glutamate-induced injury of rat spiral ganglion neurons. Mol. Med. Rep..

[90] Wright T., Gillespie L.N., O’Leary S.J., Needham K. (2016). Firing frequency and entrainment maintained in primary auditory neurons in the presence of combined BDNF and NT3. Sci. Rep..

[91] Brewer G.J. (1995). Serum-Free B27lNeurobasal Medium Supports Differentiated Growth of Neurons From the Striatum, Substantia Nigra, Septum, Cerebral Cortex, Cerebellum, and Dentate Gyrus. J. Neurosci. Res..

[92] Xie C., Markesbery W.R., Lovell M.A. (2000). Survival of hippocampal and cortical neurons in a mixture of mem and b27-supplemented neurobasal medium. Free Radic. Biol. Med..

[93] Smith T.H., Ngwainmbi J., Grider J.R., Dewey W.L., Akbarali H.I. (2013). An in-vitro preparation of isolated enteric neurons and glia from the myenteric plexus of the adult mouse. J. Vis. Exp..

[94] Wang Q., Green S.H. (2011). Functional role of neurotrophin-3 in synapse regeneration by spiral ganglion neurons on inner hair cells after excitotoxic trauma in vitro. J. Neurosci..

[95] Vincent P.F.Y., Young E.D., Edge A.S.B., Glowatzki E. (2024). Auditory hair cells and spiral ganglion neurons regenerate synapses with refined release properties in vitro. Proc. Natl. Acad. Sci. USA.

[96] Zheng J.L., Stewart R.R., Gael W.-Q. (1995). Neurotrophin-4/5 Enhances Survival of Cultured Spiral Ganglion Neurons and Protects Them from Cisplatin Neurotoxicity. J. Neurosci..

[97] Bailey E.M., Green S.H. (2014). Postnatal expression of neurotrophic factors accessible to spiral ganglion neurons in the auditory system of adult hearing and deafened rats. J. Neurosci..

[98] Needham K., Nayagam B.A., Minter R.L., O’Leary S.J. (2012). Combined application of brain-derived neurotrophic factor and neurotrophin-3 and its impact on spiral ganglion neuron firing properties and hyperpolarization-activated currents. Hear. Res..

[99] Balazs R. (2006). Trophic Effect of Glutamate. Curr. Top. Med. Chem..

[100] McDonald J.W., Johnston M.V. (1990). Physiological and pathophysiological roles of excitatory amino acids during central nervous system development. Brain Res. Brain Res. Rev..

[101] Lu C.C., Appler J.M., Andres Houseman E., Goodrich L.V. (2011). Developmental profiling of spiral ganglion neurons reveals insights into auditory circuit assembly. J. Neurosci..

[102] Lu J., Liu H., Lin S., Li C., Wu H. (2020). Electrophysiological characterization of acutely isolated spiral ganglion neurons in neonatal and mature sonic hedgehog knock-in mice. Neurosci. Lett..

[103] Feltz P., Rasminsky M. (1974). A model for the mode of action of GABA on primary afferent terminals: Depolarizing effects of GABA applied iontophoretically to neurones of mammalian dorsal root ganglia. Neuropharmacology.

[104] Akaike N., Kaneda M., Hori N., Krishtal O.A. (1988). Blockade of N-methyl-D-aspartate response in enzyme-treated rat hippocampal neurons. Neurosci. Lett..

[105] Carricondo F., Bartolomé M.V., Vicente-Torres M.A., Fernández-Pacheco P., Rodríguez T., Gil-Loyzaga P. (2002). Sensitivity to glutamate neurotoxicity in different developmental periods of the rat cochlea. Adv. Otorhinolaryngol..

[106] Wong A.B., Jing Z., Rutherford M.A., Frank T., Strenzke N., Moser T. (2013). Concurrent maturation of inner hair cell synaptic Ca^2+^ influx and auditory nerve spontaneous activity around hearing onset in mice. J. Neurosci..

[107] Frisch S.M., Francis H. (1994). Disruption of Epithelial Cell-Matrix Interactions Induces Apoptosis. J. Cell Biol..

[108] Guarnieri M., Krell L.S., Mckhann G.M., Pasternak G.W., Yama-Mura H.I. (1975). The effects of cell isolation techniques on neuronal membrane receptors. Brain Res..

[109] Allen C.N., Brady R., Swann J., Hori N., Carpenter D.O. (1988). N-MethyI-D-aspartate (NMDA) receptors are inactivated by trypsin. Brain Res..

[110] Lefebvre P.P., Weber T., Rigo J.-M., Staecker H., Moonen G., Van De Water T.R. (1992). Peripheral and central target-derived trophic factor(s) effects on auditory neurons. Hear. Res..

[111] Coleman B., Fallon J.B., Gillespie L.N., De Silva M.G., Shepherd R.K. (2007). Auditory hair cell explant co-cultures promote the differentiation of stem cells into bipolar neurons. Exp. Cell Res..

[112] Sun W., Salvi R.J. (2009). Brain derived neurotrophic factor and neurotrophic factor 3 modulate neurotransmitter receptor expressions on developing spiral ganglion neurons. Neuroscience.

[113] Ye Z.-C., Sontheimer H. (1998). Astrocytes Protect Neurons From Neurotoxic Injury by Serum Glutamate. Glia.

[114] Wood A.M., Tiwari P., Bristow D.R. (1997). Media composition modulates excitatory amino acid-induced death of rat cerebellar granule cells. Hum. Exp. Toxicol..

[115] Jonas W., Lin Y., Tortella F. (2001). Neuroprotection from glutamate toxicity with ultra-low dose glutamate. NeuroReport.

[116] Friedman L.K., Segal M. (2010). Early exposure of cultured hippocampal neurons to excitatory amino acids protects from later excitotoxicity. Int. J. Dev. Neurosci..

[117] Dux E., Oschlies U., Uto A., Kusumoto M., Siklos L., Joo F., Hossmann K.A. (1996). Serum prevents glutamate-induced mitochondrial calcium accumulation in primary neuronal cultures. Acta Neuropathol..

[118] Kume T., Kohchiyama H., Nishikawa H., Maeda T., Kaneko S., Akaike A., Noda N., Fujita T. (1997). Ether Extract of Fetal Calf Serum Protects Cultured Rat Cortical Neurons against Glutamate Cytotoxicity. Jpn. J. Pharmacol..

[119] Schramm M., Eimerlt S., Costa E. (1990). Serum and depolarizing agents cause acute neurotoxicity in cultured cerebellar granule cells: Role of the glutamate receptor responsive to N-methyl-D-aspartate. Proc. Natl. Acad. Sci. USA.

[120] Roth S., Zhang S.J., Chiu J., Wirth E.K., Schweizer U. (2010). Development of a serum-free supplement for primary neuron culture reveals the interplay of selenium and vitamin E in neuronal survival. J. Trace Elem. Med. Biol..

[121] Erdõ S.L., Michler A., Wolff J.R., Tytko H. (1990). Lack of excitotoxic cell death in serum-free cultures of rat cerebral cortex. Brain Res..

[122] Perry S.W., Norman J.P., Litzburg A., Gelbard H.A. (2004). Antioxidants are required during the early critical period, but not later, for neuronal survival. J. Neurosci. Res..

